# Seasonal Variation in 25(OH)D at Aberdeen (57°N) and Bone Health Indicators– Could Holidays in the Sun and Cod Liver Oil Supplements Alleviate Deficiency?

**DOI:** 10.1371/journal.pone.0053381

**Published:** 2013-01-08

**Authors:** Alexandra Mavroeidi, Lorna Aucott, Alison J. Black, William D. Fraser, David M. Reid, Helen M. Macdonald

**Affiliations:** 1 Musculoskeletal Research Programme, University of Aberdeen, Aberdeen, United Kingdom; 2 Norwich Medical School, University of East Anglia, Norwich, United Kingdom; University of Southampton, United Kingdom

## Abstract

Vitamin D has been linked with many health outcomes. The aim of this longitudinal study, was to assess predictors of seasonal variation of 25-hydroxy-vitamin D (25(OH)D) (including use of supplements and holidays in sunny destinations) at a northerly latitude in the UK (57°N) in relation to bone health indicators. 365 healthy postmenopausal women (mean age 62.0 y (SD 1.4)) had 25(OH)D measurements by immunoassay, serum C-telopeptide (CTX), estimates of sunlight exposure (badges of polysulphone film), information regarding holidays in sunny destinations, and diet (from food diaries, including use of supplements such as cod liver oil (CLO)) at fixed 3-monthly intervals over 15 months (subject retention 88%) with an additional 25(OH)D assessment in spring 2008. Bone mineral density (BMD) at the lumbar spine (LS) and dual hip was measured in autumn 2006 and spring 2007 (Lunar I-DXA). Deficiency prevalence (25(OH)D<25 nmol/L) was reduced in women who went on holiday to sunny destinations 3 months prior to their visit, compared to women who did not go on holidays [5.4% vs. 24.6% in Spring (p<0.001) and 3.8% vs. 25.6% in Winter (p = 0.001), respectively]. Similarly deficiency was lower amongst those who took CLO supplements compared to women that did not consume these supplements [2.0% vs. 23.7% in Spring (p = 0.001) and 4.5% vs. 24.8% in winter (p = 0.005), respectively]. There was no seasonal variation in CTX; 25(OH)D predicted a small proportion (1.8% variation) of LS BMD in spring 2007 [unstandardized β (SE): 0.039 (0.016), p = 0.017]. Seasonal variation of 25(OH)D had little effect on BMD and no effect on CTX. It appears that small increments in vitamin D (e.g. those that can be achieved by cod liver oil supplements of 5 µg/day) are sufficient to ensure that 25(OH)D is above 25 nmol/L for most people throughout the year. Similarly, holidays in sunny destinations show benefit.

## Introduction

Vitamin D has been linked with many health outcomes, including multiple sclerosis [1], cardiovascular disease [2], cancer [3] and all cause mortality [4]. The deficiency cut-off for vitamin D status [<25 nmol/L for 25-hydroxyvitamin D (25(OH)D)] was recommended with regard to preventing rickets (in children) and osteomalacia (in adults) [5]. However, the quantity required for optimum health overall, is currently hotly debated with many researchers and clinicians advocating higher intakes and a higher cut off of 25(OH)D (ranging from 50 to over 75 nmol/L) to define deficiency and insufficiency [6]–[7].

Healthy adults obtain most of their vitamin D through exposure of skin to sunlight [5] with natural dietary sources being limited [8]. Hence it might be expected that seasonality [9], latitude [10] and lifestyle would be important in vitamin D status. In addition, sunlight exposure, and thus vitamin D synthesis, is thought to be affected by Western lifestyles, which increasingly involve working indoors during daylight hours as well as residence in northern latitudes with cloudy climates [11]. As such, Scotland (higher latitude) has a higher prevalence of levels below 40 nmol/L of 25(OH)D compared to the rest of the UK [9], [12].

The aim of this study was to test the relationship between vitamin D status, assessed longitudinally, and markers of bone turnover and bone mineral density (BMD) in postmenopausal women from Aberdeen North East Scotland (57° latitude). The individual contributions on vitamin D status of a) diet (assessed by the ‘gold’ standard method of 7 day food diary [13]) including supplements that contain vitamin D such as cod liver oil and b) sunlight exposure (objectively measured by polysulphone badges) as well as holidays abroad in sunny destinations, were evaluated according to season.

## Materials and Methods

### Study Participants

**Figure 1:**
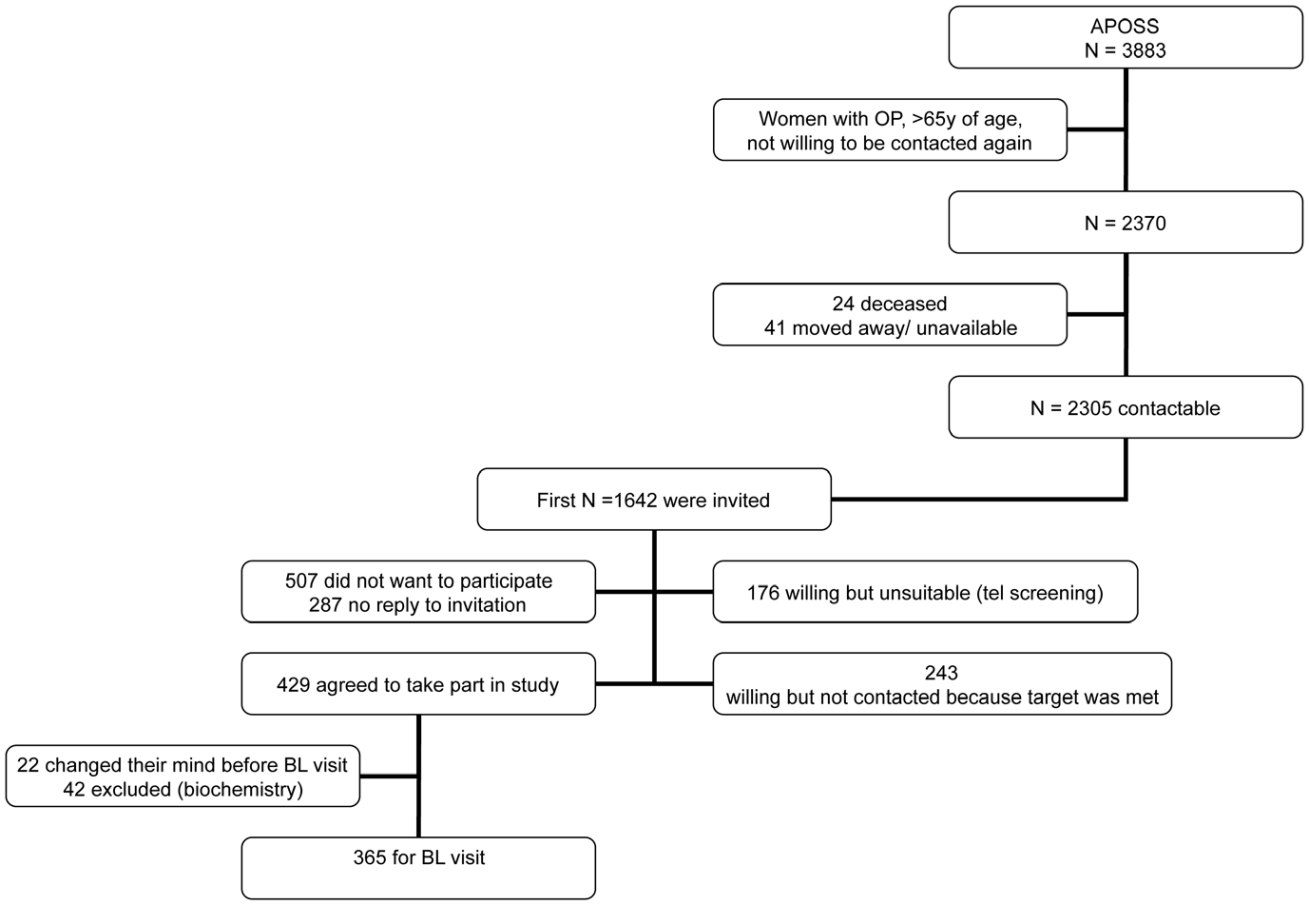
Study Flow Diagram. (APOSS: Aberdeen Prospective Osteoporosis Study, OP: Osteoporosis, BL: Baseline visit).

Subjects were postmenopausal Caucasian women recruited from a cohort of well characterised women from the Aberdeen Prospective Osteoporosis Screening Study (APOSS) [14]. The study design has been described elsewhere [12], [15]. In brief, 365 women took part in the study; a flow diagram of the study participants is shown in **Figure 1**. Women attended for 5 visits at 3 month intervals with the baseline visit being Spring 2006; 25(OH)D was also measured in Spring 2008.

Exclusion criteria included any disease or medication that could potentially affect vitamin D status. Forty two women were excluded due to abnormal routine biochemistry results obtained at their first visit (elevated liver and thyroid function tests). Women who were on vitamin D and calcium supplements were also excluded. However, we allowed women into the study if they were taking mixed multivitamins and minerals supplements and also a proportion of women (up to 20%) who were taking cod liver oil (CLO) supplements on a regular basis.

The women were weighed with a set of balance scales (Champ II Base, Ohaus, Canada) calibrated to 0.5 kg, while wearing light clothing and no shoes. Height was measured with a stadiometer (Holtain, Ltd, Crymych, United Kingdom). Socio-economic status was assessed by national deprivation scoring, based on residential postal codes, from 1 (least deprived) to 7 (most deprived) [14].

### Ethics Statement

Written informed consent was obtained for all the women and the study was approved by the North of Scotland Ethics Committee (REC reference number: 05/S0802/149). The study was registered as the Aberdeen Nutrition Sunlight and Vitamin D (ANSAViD) study at controlled-trials.com (ISRCTN96210443).

### Diet, Sunlight and Physical Activity Assessment

Nutrient intakes were assessed at baseline (Spring 2006) by a validated food frequency questionnaire (FFQ) [16]. For the remaining visits (3 mo, 6 mo, 9 mo and 12 mo) diet was assessed using the EPIC 7 day estimated food diary [17] and analysed using ‘Windiets’ (Robert Gordon University, Aberdeen, UK). The FFQ gives higher estimates of intakes compared to food diaries [18]. There was no dietary intake assessment at year 2.Vitamin D intake from supplements and cod liver oil was calculated from the frequency of use and amount of vitamin D present in the brand used as indicated by the manufacturers’ product label. Habitual physical activity (PA) assessment was carried out using the Aberdeen bone specific physical activity questionnaire (bsPAQ) [19].

UV exposure was measured using two ultraviolet sensitive polysulphone film badges worn at the lapel for 7 days at each visit. The calculation of the standard erythemal dose (SED), which is a measure of the erythemal effectiveness of a UV exposure [20], has been described elsewhere [15]. The amount of body surface area (BSA) exposed was obtained from sunlight exposure diaries that were completed daily for the duration of the study [12]. At each visit, details regarding holidays taken abroad during the preceding 3 months and use of sun-protection cream, were obtained. Women were asked at each visit whether they had been on holiday ‘to a sunny destination in the past 3 months’. If the answer was yes we asked them ‘where’ and ‘for how long’. Women were similarly asked at each study visit whether they normally wore sun protection cream and if their answer was positive what factor was the sun cream. They were also asked about the use of make-up that had UV protection.

### Biochemical Markers and Bone Mineral Density

At each visit (including the year 2 visit), fasted serum 25(OH)D was assessed by IDS enzyme immunoassay (Boldon, UK). The assay had an intra-assay CV of <16% across the working range of 6.8–380 nmol/L as defined using duplicates, calculating the CVs for all measurements and producing a precision profile. The inter-assay CV was 15.3% at 9.2 nmol/L, 7.1% at 51.5 nmol/L and 13.4% at 200.0 nmol/L. Women who had 25(OH)D below 25 nmol/L were considered to be deficient [5].

Fasted serum parathyroid hormone (PTH) was measured by Roche EIA on the E module; the intra-assay CV was <6% (precision profile) across the range 1–70 pmol/L. The inter-assay CV was 5.8% at 2.5 pmol/L, 4.9% at 7.3 pmol/L and 3.4% at 24.3 pmol/L. Fasted serum beta C-telopeptide (CTX) was measured by an Enzyme Chemiluminescent Immuno-Assay (ECLIA) supplied by Roche products Ltd (Penzberg, GmbH). The inter-assay and intra-assay CVs were less than 4% (precision profiles) across the range 0.01–2.0 µg/L. The inter-assay CV was 3.9% at 0.05 µg/L, 3.0% at 0.32 µg/L and 0.9% at 0.76 µg/L. Beta C-telopeptide and PTH were not measured at the year 2 visits. All samples, for each subject, were stored together and analyzed as a single batch.

Bone mineral density (BMD) was measured at the spine (LS) and dual hips at the autumn (2006) visit and 6 months later in spring (2007) by the same radiographer using a Lunar I-DXA scanner (GE Medical Systems Inc. Madison WI). Total (dual) hip BMD was used for subsequent analysis as the average of left and right hip BMD values. Daily phantom measurements were performed. In-house precision was obtained using 60 volunteers, showing an *in vivo* coefficient of variation of 0.56% at the lumbar spine and <0.75% at the dual hip. Percentage bone change was calculated as [(BMD spring – BMD autumn)/BMD autumn]* 100 at the lumbar spine and total hip sites.

A list of the different measurements taken at the different time points during the study can be seen in **Table 1** **.**

**Table 1:** Parameters measured at each visit throughout the study period.

Parameter measured	Spring2006	Summer 2006	Autumn 2006	Winter 2006/07	Spring 2007	Spring 2008
25(OH)D	✓	✓	✓	✓	✓	✓
PTH	✓	✓	✓	✓	✓	✓
CTX	✓	✓	✓	✓	✓	
DXA			✓		✓	
Diet	FFQ	7-day diary	7-day diary	7-day diary	7-day diary	
Dietary supplement use	✓	✓	✓	✓	✓	
Sunlight (UV badges)	✓	✓	✓	✓	✓	
BSA (Sunlight diaries )	✓	✓	✓	✓	✓	
Physical activity (bsPAQ)	✓	✓	✓	✓	✓	
Subjects’ characteristics	✓					
Questions about holidays in sunny destinations and sun protectioncream use	✓	✓	✓	✓	✓	
Questions about changes in medication and wellbeing since last visit		✓	✓	✓	✓	✓

25(OH)D: 25 hydroxy vitamin D, PTH: parathyroid hormone, CTX: beta C-telopeptide, FFQ: Food frequency questionnaire, DXA: Dual X-ray absorptiometry, BSA: Body surface area, bsPAQ: Bone specific physical activity questionnaire.

### Statistical Analysis

For the investigation of seasonal variation, the differences in mean 25(OH)D, CTX and PTH between the different seasons were tested by General Linear Model repeated measures analysis. Statistical analysis was performed using SPSS version 19. All non- normally distributed data were log-transformed before analysis.

One way ANOVA (with Scheffe post hoc analysis) was used to investigate the effect of time gap between blood sampling and last day on holidays abroad at each visit.

Mixed model analysis to include all seasons (summer 2006 to spring 2007, inclusive), was performed in SAS (SAS 9.1.3, SAS Institute Inc., Cary, NC, USA) using an unstructured covariance structure. Baseline covariates tested included age, weight, height, social deprivation category, sunscreen use, and years since menopause. Only the significant baseline covariates were used as covariates in subsequent models, with repeated measures of sunlight exposure (SED as estimated with by dosimeter badges), recent holiday abroad (yes/no), body surface area exposed and total dietary intake of vitamin D.

Linear regression was used to estimate the percentage of variance of LS and hip BMD attributed to 25(OH)D adjusted for confounders (body weight, height, smoking, social deprivation category, dietary calcium, alcohol intake, and physical activity), and to determine whether summer-winter changes in 25(OH)D, PTH and CTX or wintertime values of these variables (when 25(OH)D is lowest) could predict % BMD change.

## Results

A total of 365 women attended the baseline visit, with good retention for the 3-monthly visits ending in spring 2007 (88% attendance) and 70% returning a year later in spring 2008. The baseline characteristics are given in **Table 2**. The women that took part in our study had similar baseline characteristics (age, height, weight, BMI, smoking habits, and socioeconomic status) with the rest of the APOSS population (data not shown). One subject was suspected of suffering from primary hyperparathyroidism and was excluded from further analysis.

**Table 2:** Subject characteristics at baseline visit.

Subject characteristics	n	Mean ± SD	Min-max
Age (y)	364	62.0±1.4	59.1–65.0
Weight (kg)	352	71.7±12.9	41.0–122.0
Height (cm)	352	160.6±5.7	146.0–188.5
Metabolic component of PA (MET/h.wk)	349	71.5±66.7	11.5–194.2
Mechanical component of PA (peak scores)	349	4.5±1.6	0.0–10.0
Dietary calcium intake (mg/d)^1^	351	1040±314	243–2359
Diet and supplement calcium (mg/d)^1^	351	1042±315	243–2359
Dietary vitamin D (µg/d)^1^	351	4.3±2.5	0.5–24.4
Dietary vitamin D including supplements (µg/d)^1^	351	5.2±3.3	0.5–24.4
Energy intake (MJ/d)^1^	351	7.6±2.1	3.0–16.8
25 hydroxy vitamin D (nmol/L)	355	39.5±18.8	11.4–145.4
Parathyroid hormone (pmol/L)	355	4.9±1.5	1.9–11.4
Beta C-telopeptide (µg/L)	355	0.38±0.17	0.05–1.17
Socio-economic status^2^ (% in categories 1 to 6)	359	24, 47, 8, 11, 7, 3	

1 assessed by FFQ.

2 assessed by national deprivation scoring 1 (least deprived) to 6 (most deprived).

### Seasonal Variation of Biochemical Markers

There was a significant seasonal variation in serum 25(OH)D, with a zenith in the summer and a nadir in spring and winter (p<0.001) (**Table 3**). The percentage of population at different 25(OH)D cut off points has been reported elsewhere [21].

**Table 3:** Seasonality of serum 25(OH)D, PTH and CTX.

	N	25(OH)D*	% population	PTH**	CTX
		N	25(OH)D*	% population	PTH**
Spring 2006(Mar–May)	355	39.4 (±18.8)	21.0	5.04 (±1.59)	0.385 (±0.167)
Summer 2006(June–Aug)	335	55.7 (±20.6)	2.7	4.92 (±1.67)	0.382 (±0.163)
Autumn 2006(Sep–Nov)	329	50.6 (±21.6)	7.3	5.00 (±1.84)	0.379 (±0.161)
Winter 2006/07(Dec–Feb)	312	40.0 (±19.3)	22.0	5.10 (±1.68)	0.379 (±0.162)
Spring 2007(Mar–May)	310	40.5 (±19.5)	20.5	5.14 (±1.93)	0.376 (±0.155)
Spring 2008(Mar–May)	256	39.7 (±19.4)	24.8		

25(OH)D, 25-hydroxyvitamin D; PTH, Parathyroid hormone; CTX, beta C-telopeptide.

* Repeated measures ANOVA (Pillai’s Trace p<0.001) (variable log transformed if required).

** Repeated measures ANOVA (Pillai’s Trace p = 0.003) (variable log transformed if required).

Both p values remained significant after adjusting for multiple testing.

Periodicity in serum PTH peaked in the spring 2007 (p = 0.003), while there were no differences in CTX according to season (**Table 3**). **Table 4** shows the relationships between different markers of bone health at baseline. There was an inverse relationship between PTH and 25(OH)D (p<0.01) which was present at all visits (full data not shown). There was no association between levels of 25(OH)D and CTX either at baseline (**Table 4**) or at any subsequent visit (full data not shown).

**Table 4:** Associations between markers of bone health at baseline (Spearman correlations).

BASELINE correlations	25(OH)D	PTH	CTX	Mean total hip BMD - Autumn	Lumbar spine BMD - Autumn	Mean total hip BMD - Spring
R n	(nmol/L)	(pmol/L)	(µg/L)	(g/cm^2^)	(g/cm^2^)	(g/cm^2^)
PTH (pmol/L)	−0.255*					
	n = 357					
CTX (µg/L)	0.014	−0.016				
	n = 357	n = 357				
Mean total hip BMD (g/cm^2^) - Autumn	−0.042	0.084	−0.281*			
	n = 334	n = 334	n = 334			
Lumbar spine BMD (g/cm^2^) - Autumn	−0.062	0.071	−0.235*	0.612*		
	n = 334	n = 334	n = 334	n = 334		
Mean total hip BMD (g/cm^2^) - Spring	−0.044	0.070	−0.285*	0.993*	0.614*	
	n = 323	n = 323	n = 323	n = 321	n = 321	
Lumbar spine BMD (g/cm^2^) - Spring	−0.007	0.068	−0.262*	0.625*	0.972*	0.634*
	n = 329	n = 325	n = 325	n = 323	n = 323	n = 322

25(OH)D, 25-hydroxyvitamin D; PTH, parathyroid hormone; CTX, plasma beta C-terminal telopeptide; BMD, bone mineral density;

* P<0.01 (variable log transformed if required).

### Effect of Holidays and Cod Liver Use on 25(OH)D

Self-reported information on holidays taken in the sun (3 months prior to each visit) showed a total of 195 (62%) participants reporting taking at least one holiday in the sun during the study period, with 100 (51%) of these taking multiple holidays. Most holidays were taken in summer or autumn, with a median duration of 14 days **(** **Table 5** **).** The time interval between the date of blood sampling and the last day of the reported holiday abroad, before the sampling took place, ranged from 28d±24 (SD) in autumn 2006 to 47d±29 (spring 2007). Mean 25(OH)D did not differ between women whose blood samples were obtained less than 7 days after the end of their holidays abroad compared to 7–14 days, 14–30 and over 30 days afterwards (data not shown).

**Table 5:** Prevalence and duration of holidays abroad (in the 3 months prior to each visit) according to season.

	No holidays in the previous 3 months	On holidays in the previous 3 months	Median holiday duration
	n (%)	n (%)	days (IQR)
Spring 2006(Mar–May)	289 (80)	74 (20)	14 (7–14)
Summer 2006(June–Aug)	234 (69)	104 (31)	14 (7–14)
Autumn 2006(Sep–Nov)	221 (70)	96 (30)	14 (7–14)
Winter 2006/07(Dec–Feb)	262 (83)	52 (17)	11 (7–14)
Spring 2007(Mar–May)	251 (81)	58 (19)	14 (7–21)

**Figure 2:**
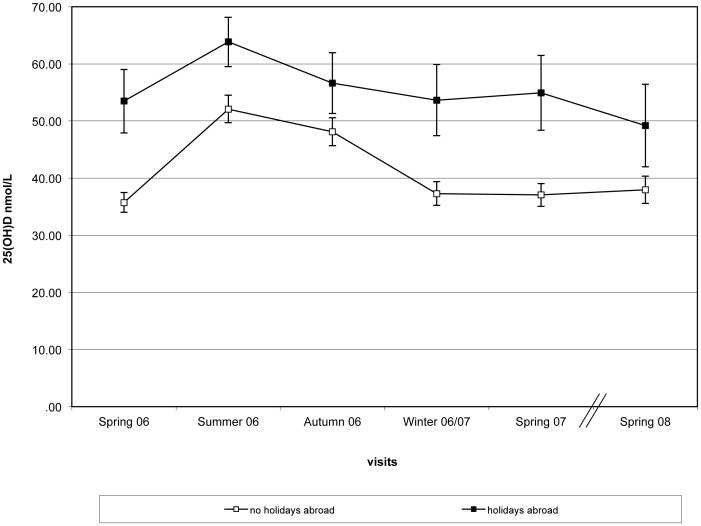
Differences in 25(OH)D between women who have had holidays abroad the preceding 3 mo from their study visits [N = 74 (spring 2006), 104 (summer 2006), 96 (autumn 2006), 52 (winter 2006/07), 58 (spring 2007), 40 (spring 2008] and those who did not (N = 289, 234, 231, 262, 251, 218, respectively). (all differences where statistically significant at p<0.001.) 25-hydroxyvitamin D (25(OH)D) (bars represent mean ± SEM) according to season/visit.

Although underlying seasonal trends were similar (**Figure 2**), the effect of holidays in sunny destinations during the 3 month period prior to blood samples being taken resulted in higher mean 25(OH)D. None of the women that reported going on holidays abroad had 25(OH)D<25 nmol/L in the summer and <7% were below this cut off point of during the rest of the year (compared to women who reported absence of holidays in sunny destinations) (**Table 6**). There were no differences in baseline characteristics (age, body weight, BMI, SED or multivitamin use) between the women who holidayed abroad and those who did not (data not shown). The low levels of deficiency (25(OH)D <25 nmol/L) that were observed in the women who went on holidays in the summer 2006 were preserved 3 months after the holiday occurred (3.2% vs. 10.8% for women who did not go on holidays, p = 0.046) but not 6 months after the initial holiday (when deficiency levels were at 29.5% vs. 30.9%, respectively). Similar results were observed for the women who chose to go on holiday in the autumn (the second most popular season to go on holidays in our population– **Table 5**). Deficiency 3 months later (in winter 2006/07) was still lower between those women who had been on holidays in a sunny destination in autumn (14.5%) compared to women who did not (30.6%, p = 0.005).

**Table 6:** Percentage of population deficient (25(OH)D <25 nmol/L) according to holidays in sunny destinations and cod liver oil use.

	Spring 2006	Summer 2006	Autumn 2006	Winter 2006/07	Spring 2007
On holidays in the previous 3 months	5.4	0.0	4.2	3.8	6.9
No holidays in the previous 3 months	24.6	3.8	8.7	25.6	23.9
P* value	<0.001	0.062	0.236	0.001	0.007
Cod liver oil use	2.0	0.0	0.0	4.5	4.5
No cod liver oil use	23.7	3.1	8.4	24.8	23.1
P* value	0.001	0.691	0.058	0.005	0.009

* chi squared test (continuity correction or Fishers exact test, where appropriate).

**Figure 3:**
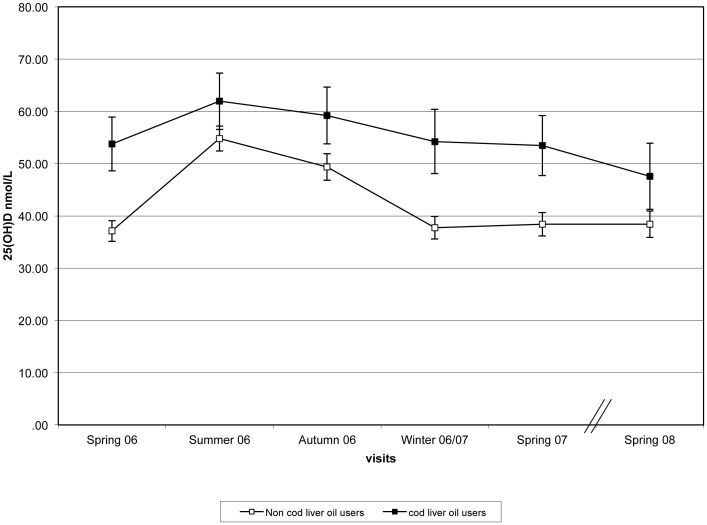
Differences in 25(OH)D between women that reported taking cod liver oil supplements [N = 50 (spring 2006), 47 (summer 2006), 46 (winter 2006/07), 44 (spring 2007), 37 (spring 2008] vs. those who did not (n = 303, 289, 285, 269, 268, 221, respectively). (all differences where statistically significant at p<0.003 except for Summer for which p<0.01.) 25-hydroxyvitamin D (25(OH)D) (bars represent mean ± SEM) according to season/visit.

Women who reported consuming CLO supplements at the baseline visit [N = 50 (spring 2006), 47 (summer 2006),46 (autumn 2006), 44 (winter 2006/07) and 37 (spring 2007)] had a higher mean 25(OH)D compared to those who did not use these supplements with the underlying seasonal trends remaining similar (**Figure 3**).

The mean total vitamin D intake (µg/d ± SD) of the women that took CLO supplements was 9.2±3.0 in spring 2006, 8.8±3.4 in summer 2006, 8.2±2.8 in autumn 2006, 8.0±2.5 in winter 2006/07, and 8.3±3.3 in spring 2007. The corresponding total vitamin D intakes for the women who did not take CLO supplements at each of the 5 visits were 4.5±2.6, 3.0±2.5, 2.8±2.2, 3.1±2.4, and 3.1±4.9 µg/d (µg/d ± SD) (p<0.001 for all differences). Intakes of vitamin D were higher for spring 2006 compared to other visits as this was assessed by FFQ instead of food diary (p<0.001 for CLO and for non-supplement users). None of the women that took CLO were deficient (i.e. 25(OH)D<25 nmol/L) in the summer/autumn, and 4.5% were below the deficiency cut off point in winter/spring 2007 (**Table 6** **)**. Again, there were no differences in baseline characteristics (age, body weight, BMI, SED or multivitamin use) between the group that took CLO supplements and the group that did not (data not shown).

### Effects of 25(OH)D Periodicity on BMD

There was no relationship between 25(OH)D and any of the BMD measurements (**Table 4**). BMD was lower by 0.7% in spring 2007 compared to the autumn 2006 at both total hip and spine sites (p<0.001) but 25(OH)D only explained 1.8% of the lumbar spine variation [unstandardized β (SE): 0.039 (0.016), p = 0.017 in the Spring 2007 measurement]. Summer-winter change in 25(OH)D, CTX and PTH were not significant predictors of % change BMD at any site and neither were winter values of 25(OH)D (when vitamin D status is at its lowest) or PTH. There was an inverse association between the CTX (bone resorption marker) and both hip and lumbar spine BMD measurements. CTX was the only variable that predicted % change BMD during winter [(2.5% of the LS variation (unstandardized β (SE): −2.692 (0.952)] and 3.0% of the total hip site variation [(unstandardized β (SE): −1.414 (0.460) (full data not shown)].

### Mixed Model Analysis

The mixed model approach with repeated measures, showed that season (summer, autumn compared to spring), recent holiday abroad (in the last 3 months) weight and BSA were significant predictors of 25(OH)D concentration (**Table 7**). Sunlight exposure, as assessed by dosimeter badges, was not a significant predictor but was included in the model, as it a known contributor of vitamin D status. The final equation including dietary vitamin D, which was significant at the 1% level was as follows: Ln 25(OH)D = 3.710+0.296 (summer) +0.328 (autumn) +0.031 (winter) –0.003 (weight in kg) +0.083 (sunblock use: yes) +0.127 (recent holiday abroad: yes) +0.002 (sunlight exposure in SED) +0.003 (BSA in %) +0.007(dietary vitamin D in µg/day). Thus for example, the model predicted that a postmenopausal woman living at 57°N, who weighed 70 kg, did not use sun cream, had not been abroad in the previous 3 months, having an average sun exposure and consuming 2.5 µg/day of dietary vitamin D, would have circulating 25(OH)D of 49.3 nmol/L in the summer. The same woman in the winter would have an estimated 25(OH)D of 35.1 nmol/L.

**Table 7:** Mixed model regression equation: all women final model.

All women: 334 subjects, 1171 observations used			
Independent variable^1^	Beta	SEM	P
Dependent variable 25(OH)D^2^	Mixed Model		
Constant	+3.710	0.105	<0.001
Season:			<0.001
Summer	+0.300	0.022	
Autumn	+0.233	0.019	
Winter	+0.031	0.016	
Spring 2007 reference	0		
Weight (kg)	−0.003	0.001	0.028
Sun block use (yes)	+0.084	0.016	<0.001
Recent holiday abroad (yes)	+0.127	0.015	<0.001
Sunlight exposure (SED)	+0.002	0.002	0.203
Body surface area (%)	+0.003	0.001	0.040
Dietary vitamin D^3^ (µg/day)	+0.007	0.003	0.009

SED: Standard erythemal dose.

1 From sunlight vitamin D (as assessed from polysulphone badges), total dietary vitamin D intake (by food diary only), recent holiday abroad, body weight, body surface area exposed to sunlight and sunblock use. Physical activity was not found to be significant predictor and not included in the final model.

2 25-hydroxyvitamin D (25(OH)D) was log transformed to obtain normal distribution.

3 Including supplements (mainly cod liver oil which adds 5 µg vitamin D a day).

All p values maintained significance after adjustment for multiple testing, except from body surface, that was borderline significant even before adjustment (p = 0.04), and body weight.

Similarly, the equation from the mixed model with repeated measures for PTH was : Ln PTH  = 1.544+0.006 (weight in kg) –0.108 Ln 25(OH)D. Only body weight and 25(OH)D were significant predictors of PTH.

## Discussion

This longitudinal study at Northerly latitude showed that despite fluctuation in vitamin D status (with a peak in summer and a nadir in winter/spring), bone resorption remained unchanged throughout the 15-month observation period (adjustment for multiple testing did not change these relationships). BMD was found to decrease by a small increment (<1%) between autumn 2006 and spring 2007. Overall serum 25(OH)D was not predictive of BMD except for a very small proportion of the variation in lumbar spine BMD in spring. A possible explanation for this might be that lifetime 25(OH)D may be more important for bone health than recent 25(OH)D measurements. In addition, our population had overall low vitamin D status and it has been suggested that levels >75 nmol/L are needed for improvements in bone health [22].

Others have reported cross-sectional data that seasonal changes in bone resorption markers are linked to seasonal changes in vitamin D status for older women in south-eastern Australia (38–39°S) [23], Cork, Ireland (52°N) [24] and Nebraska, US (41°N) [25]. We reported similar associations in our cross-sectional data [26], but this was not confirmed in the current longitudinal study where the same subjects had five consecutive serum 25(OH)D and CTX measurements at fixed 3 monthly periods. Potential explanations for this include differences in age and menopausal status (postmenopausal versus within 5 years of the menopause and 7 years younger), the bone resorption marker measured (serum CTX versus urinary free deoxypyrinoline), and sample size (365 versus 3113). However, our study sample size was adequate to determine an 8% summer-winter difference in CTX at the 89% level (p = 0.05). Alternatively the difference in study design suggests that the within-individual bone turnover variation is conserved relative to between-individual variation [15].

The 25(OH)D measures during the three spring periods were almost identical despite different weather (sunshine hours) in the preceding summer and a particular poor summer in 2007 [27].To our knowledge this is the first longitudinal study to report measures of 25(OH)D consecutively for 3 years (2006–08) during the Spring seasons, when the vitamin D status is at its lowest in the UK. This finding suggests that 25(OH)D is being conserved either by topping up from vitamin D stores in the body, or may be protected for example through vitamin D binding protein [28]. Vitamin D binding protein may behave as a serum carrier and reservoir believed to prolong the half life of circulating vitamin D and regulating its bio-availability to target tissues [29].

There was an inverse periodicity of serum PTH with 25(OH) D, significant for each season except summer, although the 25(OH)D fluctuation was not as marked in our population as reported in other studies (e.g. [23], [24], [25], [30]) and few subjects had 25(OH)D concentrations >75 nmol/L. The Northerly latitude and poor weather may explain the smaller increase of 25(OH)D during the summer, although it is then surprising that 25(OH)D did not fall to even lower concentrations in the winter. The smaller seasonal fluctuation in 25(OH)D may reduce the PTH fluctuation which in turn further reduces the CTX fluctuations. However, the concentrations of circulating PTH observed in our population were similar to others [30] (although caution should be advised when comparing studies because of differences in the analytical methods, and study designs i.e. cross-sectional vs. longitudinal data).

Ultraviolet radiation exposure from sunny holidays outside the UK appeared to alleviate the risk of deficiency (25(OH)D<25 nmol/L) for the majority of the women and this effect was preserved 3 months after the holiday occurred. It is known that travelling abroad to sunny resorts can completely change the UV dose pattern of an individual. Danish indoor workers who went on holiday for a median of 7 days received 26 SED compared with a median of 3.1 SED for the entire winter [31]. In a laboratory setting, 1.3 SED, 3 times a week for 6 weeks while wearing T-shirt and shorts, increased mean 25(OH)D from 44 to 70 nmol/L [32]. Our volunteers only wore their dosimeter badges while in Aberdeen, so we could not estimate the SED they received on holiday but the higher concentrations of 25(OH)D, compared to those who did not go on holidays abroad, show a marked effect on vitamin D status. The inclusion of sun protection cream usage as a positive predictor of 25(OH)D in the mixed model would appear to be counter intuitive, since sun protection creams are supposed to prevent UVB radiation reaching the skin. One explanation is that these women do not use sun protection cream as thickly or as often to exposed areas as would be recommended, so in fact they receive sufficient UVB radiation to be able to synthesise vitamin D.

Deficiency (25(OH)D<25 nmol/L) was also alleviated [0% (summer/autumn) and 4.5% (winter/spring)] for the majority of women who took dietary supplements containing vitamin D (cod liver oil) compared to women that only received vitamin D via food sources. Few foods naturally contain vitamin D and the addition of vitamin D from supplements (5µg or 200 IU) more than doubles overall vitamin D intakes. The effect of cod liver oil supplements in raising 25(OH)D was also observed in the UK National Diet and Nutrition Survey (NDNS) for women aged 50–64 years. They reported an association between total dietary vitamin D (including cod liver oil supplements) with 25(OH)D (r = 0.33, p<0.01) but no association between 25(OH)D and food only derived vitamin D [33]. We reported similar cross-sectional associations in the APOSS population for total dietary vitamin D intake and 25(OH) D [26]. The current study suggests that oral vitamin D intake has a significant effect in alleviating vitamin D deficiency (25(OH)D)<25 nmol/L) only when dietary supplements (cod liver oil) are included.

The strength of our data is that vitamin D was measured longitudinally in a cohort of older free living women at northerly latitude in the UK. The study was carefully done, with women attending visits at strictly controlled time intervals and included gold-standard measures of sunlight exposure and diet.

The main limitations are that our findings are observational, and not causative. The study was carried out in a small well-defined cohort of older postmenopausal Caucasian women and cannot be extrapolated to the general population. In addition, we did not measure health outcomes other than bone health. Very few individuals in our population had 25(OH)D above 75 nmol/L and as this is considered by some experts to be optimal for bone health this might pose as a limitation. The information regarding the holidays to sunny destination was self reported; we did not independently confirm the weather conditions to these holiday destinations and the dosimeter UV badges were not used abroad. As such this study has limited power to detect the exact necessary time to a sunny estimation or latitude required to alleviate 25(OH)D deficiency. It is possible that the benefits of CLO and holidays abroad are confounders as they may be confined to a healthy population (although women who went on holidays abroad did not necessarily consume CLO and vice versa and the baseline characteristics between the groups (those who went on holidays in sunny destinations or took CLO vs. those who did not) did not differ. The study was not powered to examine any potential interactive effects of holidays abroad and cod liver oil usage on vitamin D status. In addition, there may be long-term effects on bone (more than the 15 month duration of this study) that this investigation was not designed to measure (e.g. fracture rates).

### Conclusion

In conclusion, for a substantial number of women living at northerly latitudes, 25(OH)D falls below 25 nmol/L for part of the year but this did not appear to influence bone resorption and had only a very small effect on BMD which might have limited clinical significance. Whether this finding is important for long-term bone health and other related health outcomes (e.g. CVD, cancer etc) requires further interventional studies. It appears that for our postmenopausal Caucasian population small increments, as would be given in cod liver oil capsules (200 IU daily) or modest dietary supplements, are sufficient to ensure that 25(OH)D remains above 25 nmol/L for most people throughout the year. Similarly, holidays in the sun also show benefit. More may be required to maintain higher circulating 25(OH)D concentrations.
